# Association of *IRX6* rs6499755 and *HAAO* rs3816183 Polymorphisms With Hypospadias Susceptibility in Northern Chinese Han Population

**DOI:** 10.1155/genr/5775560

**Published:** 2025-06-13

**Authors:** Nan Liu, Yuping Yu, Ziying Chen, Jianbo Shu, Xiaofang Chen, Guodong Xu, Chunquan Cai

**Affiliations:** ^1^Tianjin Key Laboratory of Birth Defects for Prevention and Treatment, Tianjin Children's Hospital (Children's Hospital of Tianjin University), Tianjin 300134, China; ^2^Tianjin Pediatric Research Institute, Tianjin 300134, China; ^3^Department of Pediatrics, The First Affiliated Hospital of Nanchang University, Nanchang 330000, Jiangxi, China; ^4^Department of Urology, Tianjin Children's Hospital (Children's Hospital of Tianjin University), Tianjin 300134, China

**Keywords:** case-control study, Chinese Northern Han, *HAAO*, hypospadias, *IRX6*, single nucleotide polymorphism

## Abstract

**Background:** Hypospadias is one of the most common male congenital external genital malformation anomalies with unclear and multifactorial etiology. Our study aims to investigate whether *IRX6* rs6499755 and *HAAO* rs3816183 polymorphisms are susceptible to hypospadias in Chinese Northern Han.

**Methods:** We enrolled 113 patients with hypospadias and 182 healthy controls in the case-control study. Genotyping of single nucleotide polymorphisms (SNPs) was performed using High Resolution Melting (HRM). 113 hypospadias cases were further divided into anterior, middle and posterior subgroups for analysis. In addition, we performed a meta-analysis to evaluate the relationship in multiple populations.

**Results:** The risk allele [C] of *IRX6* rs6499755 was significantly associated with susceptibility to general hypospadias (OR = 1.547, *p*=0.01), anterior hypospadias (OR = 3.579, *p*=0.003) and posterior hypospadias (OR = 1.737, *p*=0.005). Besides, CC genotype carriers showed an increased risk of hypospadias compared with CT + TT carriers (OR = 1.832, *p*=0.026). The risk allele [T] of *HAAO* rs3816183 was associated with susceptibility to anterior/middle hypospadias (OR = 1.775, *p*=0.046). GMDR analysis revealed a significant interaction between *IRX6* rs6499755 and *HAAO* rs3816183 in the risk of hypospadias (cross-validation consistency = 10/10, testing balanced accuracy = 0.6065, *p*=0.0010). The results of meta-analysis (including 3789 cases and 9241 controls) indicated that *IRX6* rs6499755 and *HAAO* rs3816183 were significantly associated with hypospadias (both *p* < 0.00001).

**Conclusions:** *IRX6* rs6499755 and *HAAO* rs3816183 polymorphisms were associated with hypospadias in Chinese Northern Han, and there is a potential interaction between *IRX6* rs6499755 and *HAAO* rs3816183 affecting the risk of hypospadias. The meta-analysis supported the hypothesis that *IRX6* rs6499755 and *HAAO* rs3816183 were the susceptibility loci for hypospadias. Further research is needed to clarify their pathogenic mechanisms.

## 1. Introduction

Hypospadias is the second most common congenital anomalies in newborn males after undescended testis and is characterized by proximal displacement of the abnormal urethral meatus, penile curvature, and a ventrally deficient hooded foreskin [1]. According to the preoperative meatal position, hypospadias is often classified in anterior or distal (glandular, coronal, subcoronal), middle (mid-penile), and posterior or proximal (posterior penile, penoscrotal, scrotal, and perineal) [2]. The anterior/middle position is the most common form (80%∼85%) and considered mild, while the posterior cases are considered severe [3]. The prevalence of hypospadias is 5∼50 per 10,000 live births, and showing an increasing trend in worldwide [4]. Up to now, the etiology of hypospadias is still unclear. It is generally accepted that hypospadias is a highly heterogeneous condition condition, resulting from genetic predisposition and environmental factors, including parental risk, fetal risk, ethnic background, and geographic risk etc [5, 6].

Single nucleotide polymorphisms (SNPs) analysis is one of the important methods to analyze genetic susceptibility of common complex diseases. In 2014, a Genome-wide association study (GWAS) was performed on a European population and identified 17 SNPs, which were associated with hypospadias independently [7]. In 2019, all of 17 SNPs were replicated and performed in a Japanese case-control study (including 169 patients and 1148 controls), and only rs6499755 of iroquois homeobox 6 (*IRX6*) and rs3816183 of 3-hydroxyanthranilate-3,4-dioxygenase (*HAAO*) are found significantly associated with susceptibility to hypospadias [8]. In 2022, the association between *HAAO* rs3816183 polymorphisms is replicated and performed again in a cohort of Southern Han Chinese population (enrolled 577 patients and 654 controls). However, unlike the results of the Japanese study, this study based on the Chinese population only suggested an association between *HAAO* rs3816183 polymorphism and anterior/middle hypospadias, and no evidence for risk in all phenotypes [9].

It is generally accepted that the existence of significant ethnic differences in genetic factors is objective; therefore evaluating the effectiveness of each locus in different ethnic groups is necessary. This case-control study was designed to investigate whether there is any meaningful relationship between *IRX6* rs6499755 and *HAAO* rs3816183 polymorphisms with hypospadias in the Northern Han Chinese population, and try to find the corresponding risk alleles. In addition to this, we tried to further evaluate the relationship between these two SNPs and hypospadias in multiple populations through a meta-analysis, using combined data from previous studies of different races and the results of our study.

## 2. Materials and Methods

### 2.1. Ethical Compliance

Informed consent was obtained from all participants or their parents/legal guardians, and the study was approved by the Ethics Committee of Tianjin Children's Hospital (Tianjin, China).

### 2.2. Case-Control Analysis

#### 2.2.1. Case and Control Samples

We enrolled 113 patients (mean age at the first visit to hospitalization 1.2 years) with hypospadias and 182 healthy boys (mean age at inclusion 5.66 years) as controls from Tianjin Children's Hospital, a regional pediatric medical center in North China (Table S1). The diagnosis of patients was made by experienced pediatric urologists. According to the preoperative urethral meatus position, the patients were classified into three subgroups: anterior hypospadias (13 patients with glandular, coronal, or subcoronal hypospadias), middle hypospadias (20 patients with mid penile hypospadias), and posterior hypospadias (73 patients with posterior penile, penoscrotal, scrotal, or perineal hypospadias). And 7 cases could not be classified due to previously operations at other hospitals. All controls included in our study were unrelated to hypospadias, they came to hospital just for a physical examination.

#### 2.2.2. DNA Extraction and Genotyping

Human genomic DNA was extracted from peripheral blood samples of all patients and controls using a Genomic blood DNA mini kit (Beijing ComWin Biotech, Beijing, China) according to the manufacturer's protocol, and stored at −20°C for later use. Quantitative measurements of DNA concentrations were obtained using a Nanodrop Spectrophotometer (Nanodrop Technologies, Thermoscientific, Wilmington, DE, USA). The website of NCBI (https://blast.ncbi.nlm.nih.gov/Blast.cgi) was used to design primers, and Sanger sequencing was used to seek the template genotypes for each SNP locus. The genotypes of two SNPs were determined by High Resolution Melting (HRM) technology. Finally, the samples that could not be resolved clearly by HRM were sent to Sanger sequencing for accurate results.

#### 2.2.3. Statistical Analysis

SPSS software (version 26.0) was used for the data analysis. The chi-square test was used to calculate genotype and allele frequencies in cases and controls. A chi-squared goodness-of-fit test was conducted to examine the consistency between the genotype frequencies of the samples and Hardy–Weinberg equilibrium (HWE). Odds ratios (ORs) and confidence intervals (CIs) were calculated using the wild genotype of gene polymorphism locus as the reference. GMDR analysis has been used to test and verify whether there is a potential interaction between *IRX6* rs6499755 and *HAAO* rs3816183 affecting the risk of hypospadias. A *p* value of 0.05 was considered statistically significant for all tests.

#### 2.2.4. SNP Function Assessment

Predicted functional impacts of the two SNPs were analyzed by in silico prediction tools including PolyPhen-2, SIFT and REVEL.

### 2.3. Meta-Analysis

We searched three English-language databases (Web of Science, PubMed, and EMBASE) and three China-based databases (VIP, Wanfang, and China National Knowledge Infrastructure [CNKI]), to retrieve all pertinent association studies involving rs6499755 of *IRX6* or/and rs3816183 of *HAAO* and hypospadias risk published before April 15, 2022 (Table S2). The keywords were “*IRX6*,” “*HAAO*,” “rs6499755,” “rs3816183,” “single nucleotide polymorphisms,” and “hypospadias.” The data used for meta-analysis combined the collected data from previous researches and the results of our case-control study. Review Manager 5.4 software [10] was used for the meta-analysis. The heterogeneity test was undertaken by the *Q* test and *I*^2^ test. If a *p* value > 0.1 (*Q* test) as well as *I*^2^ < 50% (*I*^2^ test), heterogeneity was considered to be meaningless. In this study, we used the Mantel–Haenszel statistical method and random effect model to calculate the OR for all groups, and *p* < 0.05 suggested a significant difference.

## 3. Results

### 3.1. Results of Case-Control Analysis

#### 3.1.1. Association of *IRX6* rs6499755 and *HAAO* rs3816183 Polymorphisms With Hypospadias Susceptibility

A total of 113 patients and 182 controls were enrolled in this case-controls. The genotype frequencies of controls and patients are shown in Table 1. The frequency distribution of the *IRX6* rs6499755 and *HAAO* rs3816183 genotype in the case and control groups was consistent with HWE (*p* > 0.05). Compared with the TT genotype, the CC phenotype of *IRX6* rs6499755 was associated with the increased risk of hypospadias (CC vs. TT: OR = 2.234, 95% CI = 1.173–4.254, *p*=0.014). At the same time, the results showed that the *IRX6* rs6499755 [C] polymorphism may be associated with hypospadias susceptibility in recessive models (CC vs. CT + TT: OR = 1.832, 95% CI = 1.072–3.130, *p*=0.026). However, there was no statistically significantly associated with hypospadias risk observed in *HAAO* rs3816183 [T] polymorphism (all *p* > 0.05).

To further explore the possibility of a synergistic effect existence between the two SNPs loci, we performed a GMDR analysis. The result revealed a significant interaction between *IRX6* rs6499755 and *HAAO* rs3816183 in the risk of hypospadias (cross-validation consistency = 10/10, testing balanced accuracy = 0.6065, *p*=0.0010) (Table 2).

#### 3.1.2. Subgroups Analysis of SNPs and Hypospadias Risk According to Clinical Classifications

A total of 106 patients were classified into three subgroups (anterior, middle and posterior) based on the preoperative urethral meatus position. The risk allele [C] of *IRX6* rs6499755 was associated with increased susceptibility to overall hypospadias (OR = 1.547, 95% CI = 1.108–2.160, *p*=0.01), anterior (OR = 3.579, 95% CI = 1.468–8.724, *p*=0.003) and posterior hypospadias (OR = 1.737, 95% CI = 1.179–2.560, *p*=0.005). The risk allele [T] of *HAAO* rs3816183 had an association with increased risk of middle (OR = 2.130, 95% CI = 1.072–4.232, *p*=0.028) and anterior/middle (OR = 1.775, 95% CI = 1.005–3.135, *p*=0.046) hypospadias. Nevertheless, no significant association was found in other subgroups (all *p* > 0.05) (Table 3).

#### 3.1.3. SNP Function Assessment

SNPs were predicted through in silico prediction tools and the results are shown in Table 4. There were no scores available for SNP rs60561691. Overall, deleteriousness prediction results of rs3816183 tended to be benign.

### 3.2. Results of Meta-Analysis

Three relevant articles were obtained by searching six databases, including 3676 cases and 9059 controls [7–9], and ref.7 included two sets of unduplicated data(1006 cases, 5486 controls from Denmark, and an additional 1972 cases and 1812 controls from Denmark, the Netherlands and Sweden). We subsequently conducted a meta-analysis on two SNPs loci by combining the data of this study and the included researches. We calculated the ORs and 95% CI of the risk alleles according to the different races involved in the four studies.

**Figure 1:**
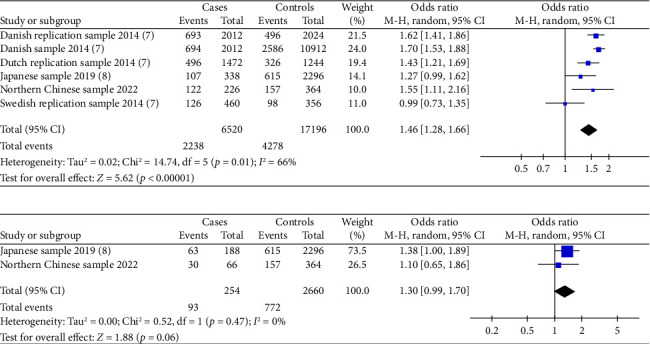
Forest plot of the association between *IRX6* rs6499755 and hypospadias. (a) Forest plot of the association between rs6499755 and general hypospadias. (b) Forest plot of the association between rs6499755 and general mild/moderate hypospadias.

Three included studies assessed the association between the risk alleles [C] of *IRX6* rs6499755 and the risk of hypospadias. The results showed a statistically significantly increased risk of hypospadias (ORs = 1.46, 95% Cl: 1.28–1.66, *p* < 0.00001) (shown in Figure 1(a)). The heterogeneity (*p*=0.01 and *I*^2^ = 66%) was considered to be moderate. There might be no significant relationship between the *IRX6* rs6499755 risk alleles [C] and anterior/middle hypospadias risk (ORs = 1.30, 95% Cl: 0.99–1.70, *p*=0.06) (shown in Figure 1(b)).

**Figure 2:**
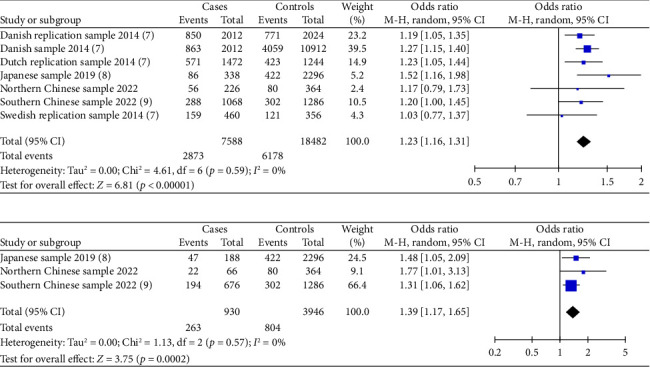
Forest plot of the association between *HAAO* rs3816183 and hypospadias. (a) Forest plot of the association between rs3816183 and general hypospadias. (b) Forest plot of the association between rs3816183 and general mild/moderate hypospadias.

Four included studies investigated the association between the risk alleles [T] of *HAAO* rs3816183 and susceptibility to hypospadias, indicating a significantly increased risk of hypospadias (ORs = 1.23, 95% Cl: 1.16–1.31, *p* < 0.00001) (shown in Figure2(a)). The heterogeneity (*p*=0.59 and *I*^2^ = 0%) was considered to be meaningless. Otherwise, we noted a significantly increased risk of anterior/middle hypospadias associated with *HAAO* rs3816183 [T] (ORs = 1.39, 95% Cl: 1.17–1.65, *p*=0.0002) (shown in Figure 2(b)). However, no significantly increased risk of posterior hypospadias was found linked to *IRX6* rs6499755 [C] and *HAAO* rs3816183 [T] (all *p* > 0.05) (shown in Figures S1 and S2).

## 4. Discussion

Hypospadias is a common congenital malformation, caused by failure of fusion of the urethral folds, endodermal differentiation, and ectodermal ingrowth in gestational 8–20 weeks, and the severity of hypospadias depends on the time of fusion failure in the embryonic period [11, 12]. The etiology of hypospadias is multifactorial and highly heterogeneous, including genetic factors and environmental factors. Environmental factors include early placental malfunction, low birthweight, maternal hypertension, pre-eclampsia, maternal intrauterine diethylstilbestrol exposure, use of intracytoplasmic sperm injection (ICSI), prolonged time-to-pregnancy, high maternal BMI, primiparity, multiple pregnancy, pre-existing maternal diabetes, maternal medication use: anti-epileptic drugs etc [13–15]. It is generally believed that hypospadias is caused by multiple genetic factors rather than a single gene [16]. To find more suspected risk loci for hypospadias, GWAS and analysis of SNPs have been increasingly used over recent years. In 2014, Geller et al. [7] identified 17 SNPs in the European population. Later, Kojima et al. [8] replicated all of these SNPs in the Japanese population found two SNPs (*IRX6* rs6499755 and *HAAO* rs3816183) associated with hypospadias. Recently, Liu et al. [9] replicated *HAAO* rs3816183 in the Southern Han Chinese population, it was also found that there was a certain correlation between the locus and hypospadias. Our study aimed to replicate the results of *IRX6* rs6499755 and *HAAO* rs3816183 using different races. Firstly, we designed a case-control analysis to assess the relationship between these two SNPs and hypospadias susceptibility in a Northern Han Chinese population. Next, we performed a meta-analysis to evaluate the relationship in multiple races, based on the results of our study and three previous studies.

Through case-control analysis, we found that *IRX6* rs6499755 was linked to an increased risk of hypospadias (OR = 1.547, *p* = 0.01), especially anterior (OR = 3.579, *p*=0.003) and posterior hypospadias (OR = 1.737, *p*=0.005), which probably through recessive models (OR = 1.832, *p*=0.026). We also observed that *HAAO* rs3816183 had an association with increased risk of anterior/middle (OR = 1.775, *p*=0.046) hypospadias, especially middle hypospadias (OR = 2.130, *p*=0.028). Besides, there was a cumulative effect between the frequency of two risk alleles and the risk of hypospadias (OR = 1.337, *p*=0.02). By performing a meta-analysis, we found a significant association between *IRX6* rs6499755/*HAAO* rs3816183 with the increased risk of hypospadias (ORs = 1.46, *p* < 0.00001; ORs = 1.23, *p* < 0.00001, independently). In the subgroup of anterior/middle hypospadias, we observed a not significantly related with *IRX6* rs6499755(ORs = 1.30, *p*=0.06) and a significantly associated with *HAAO* rs3816183 (ORs = 1.39, *p*=0.0002). Nevertheless, the further biological mechanisms of *IRX6* rs6499755/*HAAO* rs3816183 result in the increased hypospadias risks remain unclear.

*IRX6* belongs to the homeobox genes of the *Irx* family *IrxB* cluster [17]. The *Irx* family, as regulators of development, plays an important role in embryo proliferation and differentiation, including the proliferation of early embryonic cells, directed differentiation of embryonic cells, and organ formation [18, 19]. It has been reported that *Irx6* expression was observed in multiple tissues in the process of embryonic development [20, 21]. During external genitalia development of male mouse embryos, *Irx6* expresses in the ectodermal epithelium, particularly in dorsal ectodermal, and no expression was found in the ventral ectoderm adjacent to the urethral plate [8]. In both case-control analysis and meta-analysis, the results revealed that the risk allele [C] of *IRX6* rs6499755 increases the risk of hypospadias, which greatly increases the reliability of this kind of relationship. It is reported that SNP loci located in other homologous gene box families (such as *HOXA* cluster, *IRX3, IRX5*, *ZFHX3*, etc.) also increase the risk of hypospadias [7], which may suggest the focus of the future research. Different from the results of Kojima in the Japanese population, we found an association between *IRX6* rs6499755 and increased risk of general, anterior and posterior hypospadias, and no significant association with anterior/middle hypospadias in the Chinese population. However, the meta-analysis involving data from these two studies found a mild relation between the SNPs and anterior/middle hypospadias risk. Population-specific genetic backgrounds may contribute to the difference. Analysis on whole genome datasets from the 1000 Genomes Project showed that common population-specific SNPs were abundant and can cluster into regions spanning several kilobases [22]. Besides, African populations followed by the Japanese had the highest number of population-specific SNPs. However, the two SNPs studied were not in the best common population-specific SNPs-enriched windows. Considering the high similarity of origin between the Chinese and Japanese, the difference may owing to environmental factors. Moreover, GMDR analysis revealed a significant interaction between *IRX6* rs6499755 and *HAAO* rs3816183 in the risk of hypospadias, which indicated the potential linkage disequilibrium. In the future, studies with larger sample sizes and higher quality are needed, and we will further explore the mechanisms underlying the heterogeneity in phenotypic outcomes associated with the same SNP across different populations.

*HAAO* is an enzyme and widely distributed in various organs. The function of *HAAO* is catalyzing the kynurenine pathway from tryptophan to quinolinic acid [23]. Hence, it plays a role in disorders associated with altered tissue levels of quinolinic acid, such as neuronal damage [24]. Although *HAAO* has been associated with fetal malformation and death in mouse models [25], there is no significant upregulation of *HAAO* expression was observed in the developing male mouse embryos external genitalia [8]. Up to now, there is still no evidence that *HAAO* is involved in human male genital development in the embryonic period. In our study we found *HAAO* rs3816183 associated with hypospadias in both of European and Asian populations, however, we lacked the data of clinical subgroup analysis in European. In the Asian population, we observed the association between *HAAO* rs3816183 with anterior/middle hypospadias by subgroup analysis. Based on existing data, we doubted *HAAO* rs3816183 polymorphism may give rise to hinder the fusion process of the urethral folds with the midline on the ventrum of the penile shaft, especially in the middle and advanced stage by changing circulating metabolites in the kynurenine pathway in Asians [26].

Although we observed relatively positive results through our work, there are still some limitations, which remain to be improved in the future. First, the samples of the case-controls analysis are relatively small, which may result in a decrease in results veracity. A larger multicenter study is needed to determine the association between SNPs and hypospadias in the Chinese population. Besides, there are few reports on the association of these two loci with hypospadias, and no functional evidence or prediction results suggesting the SNPs can alter the molecular function or expression of the genes, causing the limitation of meta-analysis. What's more, we included only European and Asian populations with unequal distribution of data weights across populations, which may influence the results. It suggests the importance of multinational collaboration to investigate the association between SNPs and hypospadias.

## 5. Conclusions

Our study indicates that there were associations between variations of *IRX6* and *HAAO* and hypospadias risk in a Chinese Northern Han population. Furthermore, the results of the meta-analysis support identified a strong relationship between the *IRX6* and *HAAO* polymorphisms and hypospadias susceptibility in multiple populations [27]. Further researches with a larger sample size involving multiethnic (such as ethnicities from African, South American, and Australian regions, etc.) will be needed to validate the results. Besides, more in-depth and comprehensive studies are needed to explore the biological pathogenic mechanism of *IRX6* and *HAAO* leading to hypospadias.

## Figures and Tables

**Table 1:** Association between *IRX6* rs6499755 T>C/*HAAO* rs3816183 T>C polymorphism and hypospadias susceptibility.

Genotype	Cases (*n* = 113)	Controls (*n* = 182)	OR (95% CI)	*p*	*pfor HWE*
*IRX6 rs6499755*					0.26
TT	27 (23.89)	62 (34.07)	Ref	1	
CT	50 (44.25)	83 (45.60)	1.383 (0.781–2.451)	0.266	
CC	36 (31.86)	37 (20.33)	2.234 (1.173–4.254)	**0.014**	
Model					
Dominant model (CC + CT vs. TT)	86/27	120/62	1.646 (0.969–2.796)	0.640	
Recessive model (CC vs. CT + TT)	36/77	37/145	1.832 (1.072–3.130)	**0.026**	
*HAAO rs3816183*					0.55
CC	65 (57.52)	113 (62.09)	Ref	1	
CT	40 (35.40)	58 (31.87)	1.199 (0.723–1.987)	0.482	
TT	8 (7.08)	11 (6.04)	1.264 (0.484–3.304)	0.632	
Model					
Dominant model (TT + CT vs. CC)	48/65	69/113	1.209 (0.750–1.951)	0.436	
Recessive model (TT vs. CT + CC)	8/105	11/171	1.184 (0.461–3.040)	0.725	

*Note:* Values are shown as number (%); Ref, reference. *p*, *p* values and significant *p* values (< 0.05) are in bold.

Abbreviations: CI, confidence interval; HWE, Hardy–Weinberg equilibrium; OR, odds ratio.

**Table 2:** Gene-gene interaction model of *IRX6* rs6499755 and *HAAO* rs3816183 in hypospadias obtained using the GMDR method.

Model	Training bal. acc.	Testing bal. acc.	CVC	*p*
rs6499755	0.5590	0.5203	10/10	0.6230
rs3816183, rs6499755	0.6151	0.6065	10/10	**0.0010**

*Note:* Bal. Acc., balanced accuracy. *p*, *p* values and significant *p* values (< 0.05) are in bold.

Abbreviations: CVC, cross-validation consistency; OR, odds ratio; 95% CI, 95% confidence interval.

**Table 3:** Association of *IRX6* rs6499755 and *HAAO* rs3816183 with different groups of hypospadias in Chinese Northern Han population.

SNPs	Nearby gene	Minor allele	MAF		Anterior only		Anterior + middle		Middle only		Posterior only		All cases	
			Cases	Controls	OR (95%CI)	*p*	OR (95%CI)	*p*	OR (95%CI)	*p*	OR (95%CI)	*p*	OR (95%CI)	*p*
rs6499755	IRX6	C	0.540	0.431	3.579 (1.468–8.724)	**0.003**	1.099 (0.649–1.861)	0.726	0.500 (0.242–1.032)	0.057	1.737 (1.179–2.560)	**0.005**	1.547 (1.108–2.160)	**0.01**
rs3816183	HAAO	T	0.248	0.220	1.308 (0.531–3.211)	0.558	1.775 (1.005–3.135)	**0.046**	2.130 (1.072–4.232)	**0.028**	0.996 (0.627–1.585)	0.988	1.169 (0.791–1.729)	0.432

*Note:* MAF, minor (risk) allele frequency; *p*, *p* values and significant *p* values (< 0.05) are in bold.

Abbreviations: CI, confidence interval; OR, odds ratio; SNPs, single nucleotide polymorphisms.

**Table 4:** Deleteriousness prediction results of the included SNPs.

SNP	Gene	In silico prediction tools		
		PolyPhen-2	SIFT	REVEL
rs6499755	*IRX6*	NA	NA	NA
rs3816183	*HAAO*	0 Benign	1 Tolerated	0.053 Likely benign

*Note:* NA, no scores available for SNP rs60561691.

## Data Availability

The data that support the findings of this study are available on request from the corresponding authors. The data are not publicly available due to privacy or ethical restrictions.

## Supporting Information

Supporting Information 1 Table S1: Demographic characteristics of the study subjects.

Supporting Information 2 Table S2: The characteristics of the included studies in the meta-analysis.

Supporting Information 3 Figure S1: Forest plot of the association between *IRX6* rs6499755 and posterior hypospadias.

Supporting Information 4 Figure S2: Forest plot of the association between *HAAO* rs3816183 and posterior hypospadias.
