# Supplementary material for: Association of IRX6 rs6499755 and HAAO rs3816183 Polymorphisms With Hypospadias Susceptibility in Northern Chinese Han Population
Source: Genet Res (Camb). 2025 Jun 13;2025:5775560. doi: 10.1155/genr/5775560 (PMC12181654; doi:10.1155/genr/5775560)
Supplement: Supporting Information 1 — Table S1: Demographic characteristics of the study subjects. [file 5775560.f1.docx]

**Table S1.** Demographic characteristics of the study subjects.

| Characteristics | Discovery stage | |
| --- | --- | --- |
|  | Cases (N=113) | Controls (N=182) |
| Age (year)^a^ |  |  |
| Median (P_25_, P_75_) | 0.92(0.5, 1.0) | 5.0(3.0, 8.0) |
| Hypospadias classification^b^ |  |  |
| Anterior | 13(11.5%) | 0 |
| Middle | 20(17.7%) | 0 |
| Posterior | 73(64.6%) | 0 |
| unknow | 7(6.2%) | 0 |

^a^Age at the first visits to the hospitalization for cases or at recruitment for controls;

^b^Hypospadias cases were classified into anterior (glandular, coronal, and subcoronal), middle (mid penile), or posterior (posterior penile, penoscrotal, scrotal, and perineal) according to the abnormal location of the urethral opening.
