# Supplementary material for: Association of IRX6 rs6499755 and HAAO rs3816183 Polymorphisms With Hypospadias Susceptibility in Northern Chinese Han Population
Source: Genet Res (Camb). 2025 Jun 13;2025:5775560. doi: 10.1155/genr/5775560 (PMC12181654; doi:10.1155/genr/5775560)

Table S2 The characteristics of the included studies in the meta-analysis

| Study (year) | Region, Country | Sample size case group | Sample size  control group | Male  (control，%) | Mean age (years), case group | Mean age (years), control group | Inclusion Criteria |
| --- | --- | --- | --- | --- | --- | --- | --- |
| Geller et al. (2014) [7] | Danish | 1006 | 5486 | 43.6 | NA | NA | 1.Underwent surgery for hypospadias  2.SNP of *IRX6* and *HAAO* genotyping was performed |
| Geller et al. (2014) [7] | Danish* | 1006 | 1012 | NA | NA | NA | 1.Diagnosis of hypospadias  2.SNP of *IRX6* and *HAAO* genotyping was performed |
| Geller et al. (2014) [7] | Dutch* | 736 | 622 | NA | NA | NA |  |
| Geller et al. (2014) [7] | Swedish* | 230 | 178 | NA | NA | NA |  |
| Kojima Y, et al. (2019) [8] | Japan | 169 | 1148 | 100 | 3.7 | NA | 1.Undergoing repair of hypospadias  2.SNP of *IRX6* and *HAAO* genotyping was performed |
| Liu Y, et al. (2022) [9] | Southern China | 534 | 643 | 100 | NA | NA | 1.Diagnosis of hypospadias  2.SNP of *HAAO* genotyping was performed |
| Nan L, et al.  （This study） | Nouthern China | 113 | 182 | 100 | 1.2 | 5.66 | 1.Diagnosis of hypospadias  2.SNP of *IRX6* and *HAAO* genotyping was performed |

Abbreviations: *, the additional complete replication set of Ref.7; SNP, single nucleotide polymorphism; NA, not available.

**Literature Search Strategy**

The databases searched by two independent coworkers included three English databases ( Web of Science, PubMed, and EMBASE) and three Chinese databases [VIP, Wanfang, and China National Knowledge Infrastructure (CNKI)] for case-control analysis or Genome-wide association study published from the inception of the databases to April 15, 2022. The search strategies were (“hypospadias” OR “genitourinary system abnormality” OR “penile abnormality” OR “urethra abnormality”)AND (“*IRX6*” OR “Iroquois homeobox 6” OR “rs6499755” OR “*HAAO*” OR “3-hydroxyanthraminobenzoat-3, 4-dioxygenase” OR “rs3816183” OR “single nucleotide polymorphism”OR “SNP” OR “genetic susceptibility”) AND (“case-control” OR “genome-wide association study”) . There was no any restriction on the language, but studies carried out on animals were excluded. Reference tables of all meta-analyses, involved reviews, and obtained papers were manually searched to check for studies that had not appeared previously. Inconsistency was resolved by consensus.

**Inclusion and exclusion criteria**

Inclusion criteria in the meta-analysis were the following:(1) the children of case group were diagnosed as hypospadias, with or without surgery; (2) the children of control group were healthy children, who did not have hypospadias;(3) the analysis of *IRX6* rs6499755 and *HAAO* rs3816183 polymorphisms was performed for both groups; (3) Case-control analysis or Genome-wide association study.

Exclusion criteria in our research were as follows: (1) Not Case-control analysis or Genome-wide association study, including abstracts only, case reports, reviews, conference proceedings, animal studies and non-clinical studies; (2) No available date, for example studies with insufficient data for estimating the standard mean difference (SMD) and 95% confidence interval (CI); (3) Duplicated studies reporting, repeated data and analysis.

**Study Selection and Data Extraction**

According to the inclusion and exclusion criteria, two authors screened titles and abstracts independently to identify potential articles. Another two authors selected and decided the final studies included in the analysis by screening the full texts of the potential articles. Two researchers evaluated study details from all included studies and extracted data using standardized forms. The data included following items: first author's name, year of publication, ethnicity of the studied population, definition of cases, male percentage and mean ages in case and control groups, sample size of case and control groups, genotype distributions. Disagreements arising during this process were resolved and reached consensus by collective discussion.

**Quality Assessment and Risk of Bias**

The quality and risk of bias critical appraisals of included studies was assessed independently with conflicts resolved through discussion by three researchers. Review Manager 5.4 software was used for data analysis. The correlation between rs6499755 and rs3816183 polymorphisms and hypospadias was expressed by OR value and 95% CI, P<0.05 was statistically significant. The heterogeneity test was undertaken by Q test and I^2^ test. If a P-value > 0.1(Q test) as well as I^2^ < 50% (I^2^ test), heterogeneity was considered to be meaningless, therefore a fixed effect model would be used. In contrast, a random effect model was used. In this study, random effect model was used.


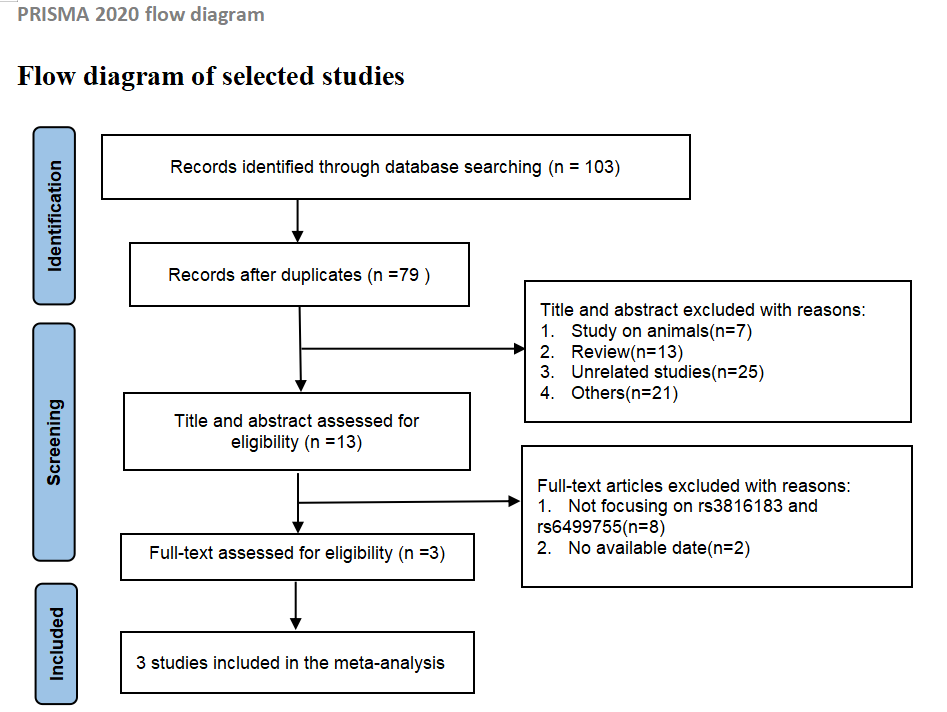

Supplement: Supporting Information 2 — Table S2: The characteristics of the included studies in the meta-analysis. [file 5775560.f2.docx]
