# Supplementary figures and images for: Association of IRX6 rs6499755 and HAAO rs3816183 Polymorphisms With Hypospadias Susceptibility in Northern Chinese Han Population
Source: Genet Res (Camb). 2025 Jun 13;2025:5775560. doi: 10.1155/genr/5775560 (PMC12181654; doi:10.1155/genr/5775560)

IRX6 rs6499755 T>C

Posterior vs controls

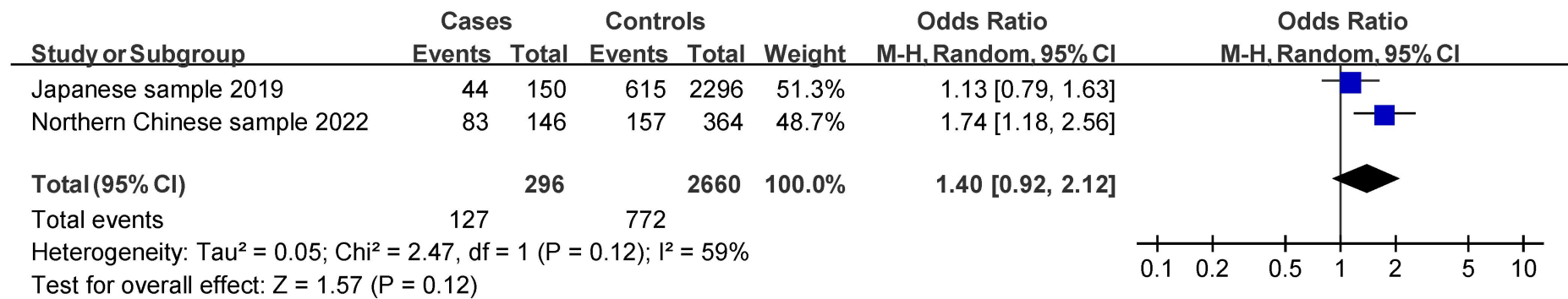

Supplement: Supporting Information 3 — Figure S1: Forest plot of the association between IRX6 rs6499755 and posterior hypospadias. [file 5775560.f3.pdf]

\_ HAAO rs3816183 T>C

Posterior vs controls

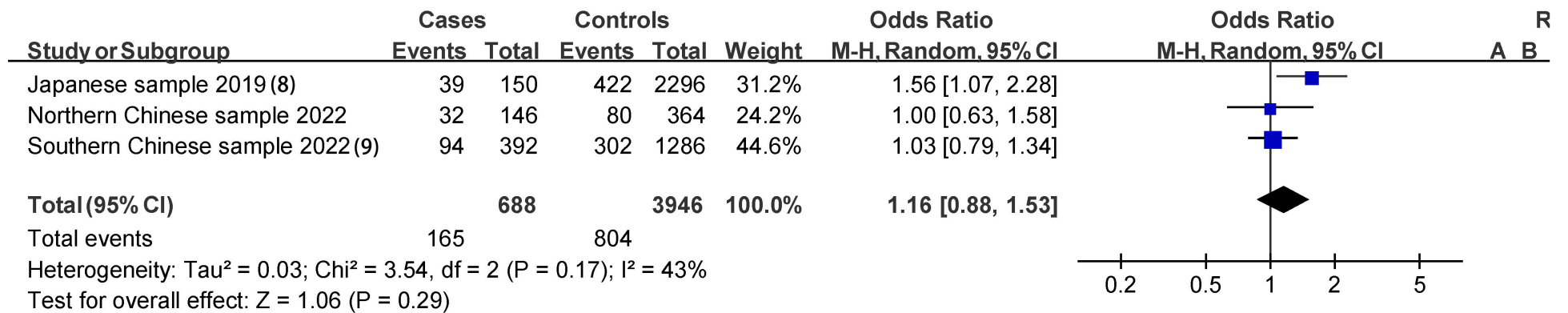

Supplement: Supporting Information 4 — Figure S2: Forest plot of the association between HAAO rs3816183 and posterior hypospadias. [file 5775560.f4.pdf]
